# Bow-tie architecture of gene regulatory networks in species of varying complexity

**DOI:** 10.1098/rsif.2021.0069

**Published:** 2021-06-09

**Authors:** Gourab Ghosh Roy, Shan He, Nicholas Geard, Karin Verspoor

**Affiliations:** ^1^ School of Computer Science, University of Birmingham, Birmingham B15 2TT, UK; ^2^ School of Computing and Information Systems, University of Melbourne, Melbourne, Victoria, Australia

**Keywords:** bow-tie architecture, gene regulatory network, biological complexity, dynamical properties

## Abstract

The gene regulatory network (GRN) architecture plays a key role in explaining the biological differences between species. We aim to understand species differences in terms of some universally present dynamical properties of their gene regulatory systems. A network architectural feature associated with controlling system-level dynamical properties is the bow-tie, identified by a strongly connected subnetwork, the core layer, between two sets of nodes, the in and the out layers. Though a bow-tie architecture has been observed in many networks, its existence has not been extensively investigated in GRNs of species of widely varying biological complexity. We analyse publicly available GRNs of several well-studied species from prokaryotes to unicellular eukaryotes to multicellular organisms. In their GRNs, we find the existence of a bow-tie architecture with a distinct largest strongly connected core layer. We show that the bow-tie architecture is a characteristic feature of GRNs. We observe an increasing trend in the relative core size with species complexity. Using studied relationships of the core size with dynamical properties like robustness and fragility, flexibility, criticality, controllability and evolvability, we hypothesize how these regulatory system properties have emerged differently with biological complexity, based on the observed differences of the GRN bow-tie architectures.

## 1. Introduction

A key objective of comparative biology is explaining biological differences between species. Gene regulation plays a critical role in explaining such organismal differences [1]. Gene regulatory networks (GRNs) [2] are networks where edges connect regulator nodes, such as transcription factors (TFs), to target nodes. A GRN is a model of the gene regulatory system that controls the development, function and pathology of organisms, and hence its analysis is extremely important. Study of GRN structure and how it varies between species can provide insights into how changes in gene expression, underlying divergence in phenotypes, occur between species [3]. Differences in GRN architectural organization are considered the reason for differential dynamic regulatory behaviour between eukaryotic yeast (*Saccharomyces cerevisiae*) and prokaryotic bacteria [4]. Comparison across multiple eukaryotes reveals a common architectural feature of the GRN—a scale-free topology, but with species-specific characteristics likely to produce species-specific phenotypes [5]. So it is vital to analyse the differences in GRN architecture to understand differences between species.

Differences between species are exhibited at various levels like anatomy, physiology and behaviour. One approach to understanding the differences between species is looking at differences in universally present dynamical regulatory system properties. Complex biological systems display some inherent system-level dynamical properties like robustness, which are related to the network dynamics and supported by specific network architectural features [6]. Understanding the emergence of these properties is important for understanding the functioning and pathology of organisms, and can support effective systems-based therapy design for critical diseases like cancer [7]. We want to investigate how these important dynamical system properties, ubiquitous in the context of gene regulation, have evolved differently between different species. For this purpose, analysing the architecture of their GRNs becomes crucial.

**Figure 1.**
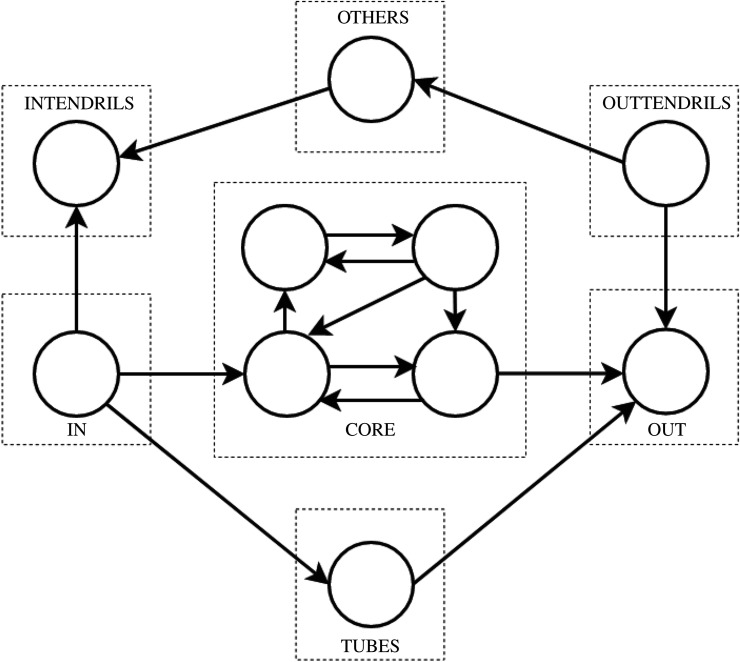
An example of a bow-tie architecture with the largest strong component (LSC) core layer. The circles represent nodes and the arrows represent edges. The different bow-tie layers are denoted by dashed boxes.

A network architecture associated with important dynamical properties like robustness, flexibility, controllability and evolvability [8] is the *bow-tie*. The bow-tie architecture has been observed in various network types, including information networks [9], internet protocol networks [10], neural networks [11] and biological networks like metabolic [12] and signalling networks [13]. The formal definition of the bow-tie architecture in a directed graph is given in terms of a strongly connected component (SCC) [14]. An SCC is a subnetwork in which every node is connected to every other node. The largest of these, the largest strong component (LSC) in the network is defined to be the bow-tie core layer [9,12]. The LSC core lies between the in layer and the out layer. As presented in figure 1, the rest of the nodes in the network are categorized into remaining layers of the bow-tie—intendrils, outtendrils, tubes and others.

Researchers have previously shown the existence of a bow-tie architecture in GRNs of some eukaryotes, with the LSC core being the only non-trivial (consisting of more than one node) strong component. For example, the work in [4] demonstrates that a bow-tie architecture with one large strongly connected core is observed in the yeast (*S. cerevisiae*) GRN’s dynamical backbone, defined as a subgraph of computationally relevant dependencies. However, the authors observed a top-down hierarchy but not a bow-tie structure in the dynamical backbones of bacteria *Bacillus subtilis* and *Escherichia coli* GRNs. The other example is that condition-specific TF–TF regulatory networks of the plant *Arabidopsis* (*A. thaliana*) in six tested experimental conditions exhibit a bow-tie architecture with one non-trivial distinct LSC core [15]. The authors in [15] additionally speculate that such an architecture might be prevalent in other eukaryotic species. However, the existence of bow-tie architectures and the quantification of their characteristics across GRNs in species of a wide range of biological complexity have not yet been addressed.

In this paper, we investigate the existence of the bow-tie architecture in GRNs of a number of well-studied species, which cover a wide range of biological complexity. Complexity is defined on the basis of the number of different cell types in each organism [16]. We make use of transcriptional regulation information from public databases to construct GRNs for prokaryotes to unicellular eukaryotes to different phyla of multicellular species. Here we analyse general GRNs, which are not specific to any particular context like experimental condition or cell type, and cover a high percentage of the species total genes, to look for these global regulatory architectures in different species. We apply architectural decomposition [14] to these GRNs to find a bow-tie architecture with an LSC core. To understand the potential biological significance of observed trends, we build on studied associations of the bow-tie architecture with dynamical system properties. The bow-tie core size, both absolute (number of nodes or regulators) and relative (number of nodes or regulators relative to the corresponding total number in the network), is considered to be a vital aspect of the network architecture [17], as it is related to important dynamical system properties [8]. Such properties include robustness and fragility, flexibility, criticality, controllability and evolvability, universally present in complex gene regulatory systems. The bow-tie analysis in our work is aimed at the novel objective of demonstrating how differences in this particular GRN architectural feature can provide a useful perspective from which to assess differences between species of widely varying complexity in terms of these dynamical gene regulatory system properties.

Our main findings are summarized as follows:

— The GRNs of diverse species display a bow-tie architecture with a distinct LSC core layer. The bow-tie architecture of these GRNs is a characteristic feature, which can not be explained by chance.
— The size of the bow-tie core, relative to both the total number of nodes and the total number of regulators in the GRN, generally increases with the complexity of the species, suggesting a possible relationship between biological complexity and how dynamical regulatory system properties have emerged differently between species.

## 2. Material and methods

### 2.1. GRN extraction

In our study, we have selected some species covering a wide range of biological complexity, for which the GRNs are readily available from public data sources. These different sources for GRNs have been created and managed by curators using methodologies differing slightly or even widely. However, in our analysis we need a common ground for GRN comparison. Our objective has been to use the GRN extraction criteria that provide the best possible ground of subsequent comparative analysis, in terms of completeness and similarity.

GRNs can capture several forms of regulatory interactions. In the extracted networks of our analysis, the regulators are TF genes, where TFs can also refer to factors classified as TFs in the data, like sigma factors in prokaryotes or co-factors or chromatin remodelling factors in eukaryotes. The target genes can represent TF, microRNA, small RNA or other genes whose transcription is controlled by these regulators. Like in [18], we have excluded the regulatory interactions where the source genes represent non-coding RNAs like bacterial small RNAs or microRNAs. However unlike [18], we have incorporated the interactions where the regulators are TF genes which regulate the transcription of non-coding RNA target genes. We have aimed to use the most unique gene identifiers present in the data source and extract only the regulatory interactions with valid identifiers. Where possible, a complex/operon/heteromer is to be included in the network as its individual genes. For ease of use, we have selected only the TF–target gene interactions available in the data sources, when in some sources there can be additional related information like that of TF binding sites, promoters or gene expression correlation. The GRNs in our study are assumed to be general, and not specific to any particular experimental condition or cell type.

One important aspect in extracting the GRNs is the type and reliability of evidence associated with the interactions. An interaction can be experimentally validated or computationally predicted, and the interaction can be ranked based on the reliability of the evidence. All these different data sources use their own set of criteria for defining these interaction properties, and in some cases that information is not available. Choosing the strictest possible threshold on these interaction properties could lead to incomplete information for some species, which is not suitable for a reliable analysis. In our study, we extract all interactions with any evidence. Although extracting interactions without a threshold might lead to false positive edges, it eliminates the variability of analysis caused by different selections of threshold. We have excluded interactions which are categorized as indirect in the data source.

Completeness of the data is an important factor while extracting GRNs. We have addressed the issue of incompleteness of the data sources by only considering extracted GRNs with coverage of more than 50% of the species total genes. These total gene (protein + RNA) numbers for all species were obtained from the Kyoto Encyclopedia of Genes and Genomes (KEGG) Genome database [19]. For some species, there are multiple different data sources. To finally have one data source per species in our analysis, we have used the one with the highest percentage of the total genes in the species. The data sources and the corresponding extraction criteria for GRNs of well-studied species selected for our architecture analysis are listed in table 1. We believe that these network extraction criteria give us the most optimally complete and fair ground of comparison possible across GRNs of several species from different sources.

**Table 1.** GRN data sources selected for analysis.

species	data source	extraction criteria	% total genes
*E. coli*	RegulonDB	all TF–target gene and sigma factor–target gene interactions	54
yeast	YTRP	all direct TF–target gene interactions with binding evidence in the shortest pathway connecting a TF–target gene pair with expression evidence	80
*Arabidopsis*	AtRegNet	all direct TF–target gene interactions with TF and target gene name and locus specified	57
*Drosophila*	DROID	all TF–target gene interactions	81
mouse	RegNetwork	all TF–target gene interactions	73
human	RegNetwork	all TF–target gene interactions	99

The extraction criteria specific to each data source are given with the percentage of species total genes (protein + RNA) in the extracted GRN (denoted as % total genes, rounded to whole numbers). For the list of data sources not selected for analysis, see the electronic supplementary material.

Among the selected GRNs, *E. coli* K-12 GRN was extracted from the RegulonDB database [20]. The GRN contains TF–target gene and sigma factor–target gene interactions curated from literature with different ranks of experimental evidence, including some which are predicted. For yeast (*S. cerevisiae*), the yeast Transcriptional Regulatory Pathway (YTRP) database [21] was used, which consists of curated interactions with evidence of either TF–target gene binding or target gene expression variation on perturbation of TF, or both. We extracted the TF–target gene direct pairs with experimental binding evidence in the shortest regulatory pathway connecting a TF and a target gene with expression evidence. The *A. thaliana* GRN consists of different ranks of direct TF–target gene interactions obtained from the *A. thaliana* regulatory network (AtRegNet) database available on Arabidopsis Gene Regulatory Information Server (AGRIS) [22]. The GRN of *Drosophila melanogaster* consists of TF–target gene interactions with experimental evidence of the TF binding to the gene and regulating its transcription, or only binding evidence, obtained from the Drosophila Interactions Database (DroID) [23]. The data source used for mouse (*Mus musculus*) and human (*Homo sapiens*) GRNs is RegNetwork [24]. These extracted GRNs have TF–target gene interactions with different ranks of experimental or predicted evidence. These GRNs have observed percentages of the species total genes higher than the GRNs from other data sources for these two species (see electronic supplementary material).

### 2.2. Characterization of species complexity

In this section, we describe how we have characterized the notion of biological complexity in our analysis. The complexity of an organism can be defined in many ways, like genomic complexity [25] and phenotypic complexity [26]. In our study, the six species for which GRNs are selected are arranged in an order of complexity defined on the basis of their number of cell types [16]. A widely accepted precise definition of a cell type is not available, and researchers have used mostly morphological characteristics to differentiate between types [27]. However, the stable equilibrium states or gene expression patterns of GRNs are viewed to be corresponding to gene expression profiles associated with each cell type [28]. So we believe that this definition of biological complexity is relevant in our study where we analyse GRNs of different species.

We have used the knowledge about the number of cell types of different species from the literature [16,27]. When the data for a particular species were not available in the used sources, we have used the maximum number of cell types observed in the major group the species belongs to. *Escherichia coli* is the simplest organism in our study as it is a prokaryotic eubacteria, which have a maximum of two cell types. Unicellular eukaryote yeast is ranked next in complexity with maximum three cell types in *Saccharomyces* genus. For the phyla of *Arabidopsis* and *Drosophila*, the number of maximum observed cell types are 44 and 69, respectively, and hence they are arranged in that order. The next more complex species is mouse with 102 cell types. Finally, we have the species human with 411 cell types including 145 types of neurons [29]. We have used this order of complexity in presenting all our results.

### 2.3. Bow-tie architecture decomposition

To analyse the architecture of GRNs, we have used the strongly connected component based bow-tie architecture decomposition [14]. In some other definitions, the bow-tie network structure needs to resemble an hourglass, with the intermediate core smaller than the input and output layers [30]. However, this bow-tie definition, as used in our work, does not have this particular requirement. The details of the decomposition are given as follows. Let a directed network *G* be represented with a set *V* of vertices and a set *E* of edges. A destination node is defined to be reachable from a source node if there is a directed path from the source to the destination node. This definition of reachability (to or from) is extended to sets of nodes if there is a path to or from at least one node in that set. A strongly connected component is a set of nodes where every node is reachable from every other node in the set. By definition, every single node is a trivial strongly connected component. The bow-tie decomposition of the network *G* = (*V*, *E*) with the largest strong component (LSC) defined to be the core decomposes the network (figure 1) into these seven different layers or sets of nodes:

1. CORE=LSC
2. IN={v∈V−CORE | CORE is reachable from v}
3. OUT={v∈V−CORE | v is reachable from CORE}
4. INTENDRILS={v∈V−CORE | v is reachable from IN and OUT is not reachable from v}
5. OUTTENDRILS={v∈V−CORE | v is not reachable from IN and OUT is reachable from v}
6. TUBES={v∈V−CORE−IN−OUT | v is reachable from IN and OUT is reachable from v}
7. OTHERS=V−CORE−IN−OUT−INTENDRILS−OUTTENDRILS−TUBES.

The bow-tie decomposition is performed using algorithm 1 DFS_*G*_(*v*) represents the set of nodes obtained from a depth-first search starting at vertex *v* in network *G*. *G*^*T*^ refers to the network that is obtained by reversing the direction of every edge in *G*.

**Algorithm 1.** Bow-tie network decomposition algorithm based on the largest strong component (LSC) as core layer.

Set CORE=LSC. $\mathrm{Selecta}\;v\in \mathrm{CORE}.\;\mathrm{IN}={\mathrm{DFS}}_{G^{T}}(v)-\mathrm{CORE}.$ $\mathrm{Selecta}\;v\in \mathrm{CORE}.\;\mathrm{OUT}={\mathrm{DFS}}_{G}(v)-\mathrm{CORE}.$ **foreach** v∈V−CORE−IN−OUT **do** $IRV=(\mathrm{IN}\cap {\mathrm{DFS}}_{G^{T}}(v)\neq \phi ).$ $VRO=(\mathrm{OUT}\cap {\mathrm{DFS}}_{G}(v)\neq \phi ).$ **if** IRV and not VRO **then** v∈INTENDRILS. **else if** not IRV and VRO **then** v∈OUTTENDRILS. **else if** IRV and VRO **then** v∈TUBES. **else** v∈OTHERS. **end if** **end foreach**

### 2.4. Null model construction

We compared the GRNs of different species with their randomized counterparts in which the number of nodes and the degree at each node are preserved. Similar to the approach in [4], we generate these random networks. The autoregulatory edges of the original GRN are preserved separately because they do not affect the bow-tie layer definitions. This random generation process starts with the other non-autoregulatory edges in the original GRN forming the initial edge list. A pair is selected randomly from this list and their end nodes are swapped. If any of these new edges lead to self-loops or multiple edges, this swap operation is not performed for that pair. After trying the swap operation on every distinct pair in the edge list for an iteration, the algorithm in the next iteration repeats the process on the new edge list, consisting of edges from the pairs which could not be swapped. To make the process efficient on one hand and to have enough iterations for many swap operations to possibly occur on the other, we chose the number of iterations to be 10. There can be some edges whose end nodes are not swapped with another edge even after the 10 iterations. There are other ways of generating these null model networks, here we have used this simple and fast method for our analysis. 1000 such random networks were generated independently for each GRN.

## 3. Results

**Figure 2.**
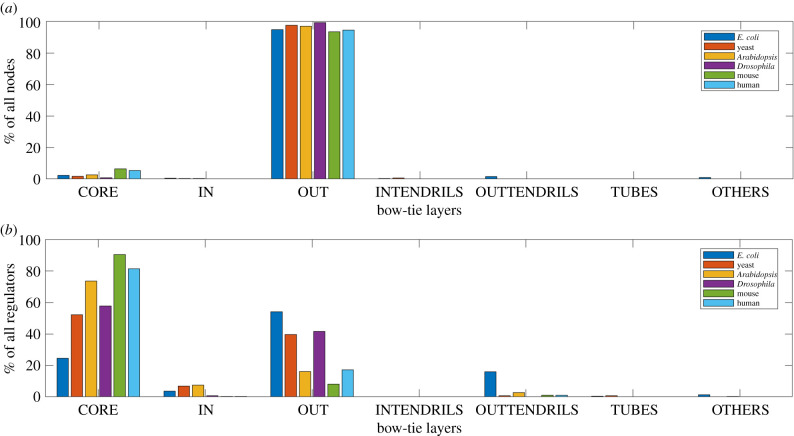
Bow-tie decomposition of GRNs. (*a*) Distribution of nodes in different bow-tie layers of GRNs in different species. (*b*) Distribution of regulators in different bow-tie layers of GRNs in different species. The core consists of a substantial percentage of all regulators. The relative core size generally increases with species complexity.

In this section, we present the results of applying the bow-tie architecture decomposition (described in §2.3) on the selected GRNs of six species of varying complexity. Table 2 shows the number of nodes and regulators in each of the bow-tie layers in these GRNs, where regulators are nodes with at least one outgoing edge in the extracted GRN. We present the relative sizes of these layers with respect to all nodes and all regulators in the network in figure 2*a*,*b*, respectively.

**Table 2.** Bow-tie decomposition of GRNs in different species.

layer		*E. coli*	yeast	*Arabidopsis*	*Drosophila*	mouse	human
all	Edges	7348	16 032	670 771	157 462	120 579	171 946
	Nodes	2381	5124	16 427	12 323	18 916	22 121
	Regs	220	159	573	149	1328	1456
core	Nodes	54	83	422	86	1203	1187
	Regs	54	83	422	86	1203	1187
2nd LSC	Nodes	3	2	1	2	3	3
	Regs	3	2	1	2	3	3
in	Nodes	8	11	43	1	3	3
	Regs	8	11	43	1	3	3
out	Nodes	2257	5003	15 943	12 236	17 670	20 901
	Regs	119	63	92	62	108	249
intendrils	Nodes	7	25	2	0	23	13
	Regs	0	0	0	0	0	0
outtendrils	Nodes	35	1	15	0	14	15
	Regs	35	1	15	0	14	15
tubes	Nodes	1	1	0	0	0	0
	Regs	1	1	0	0	0	0
others	Nodes	19	0	2	0	3	2
	Regs	3	0	1	0	0	2

The regulators (denoted as Regs) are the nodes which have at least one outgoing edge in the extracted GRN. The second LSC refers to the next largest strong component separate from the LSC core.

From table 2, we observe that for all these GRNs there is a non-trivial LSC substantially larger than the second LSC. For example in *E. coli* GRN, the LSC consists of 54 nodes compared to a three-node second LSC, and the difference between the two are larger for other species. In all these GRNs, this LSC is the distinct core of the bow-tie, located between a smaller in layer and a larger out layer. As evident from figure 2*b*, the non-trivial core which consists only of regulators by definition, consists of a substantial percentage of all regulator nodes, specially for eukaryotes (greater than 40%). We can therefore conclude that a bow-tie architecture with one distinct LSC core exists in the GRNs of all these species of varying complexity.

The GRN bow-tie architecture observed in our results has some important differences between species. Through the arrangement of species in an increasing order of biological complexity from *E. coli* to human, in table 2 and figure 2, we observe the relationship of the bow-tie core size with this biological complexity. Since we are comparing differently sized GRNs, we have examined the variation of relative core size. This variation is clear in figure 2*a* and especially in figure 2*b*. The relative core size roughly increases as species complexity increases. This increase in percentage of network regulators in the bow-tie core in more complex organisms comes at the cost of a roughly decreasing percentage of regulators in the in and the out layers, as can be observed in figure 2*b*. Based on our observations, we can conclude that structurally the core size is a key differentiating factor in the bow-tie GRN architecture of different species, with a relatively larger core observed in more complex organisms.

**Figure 3.**
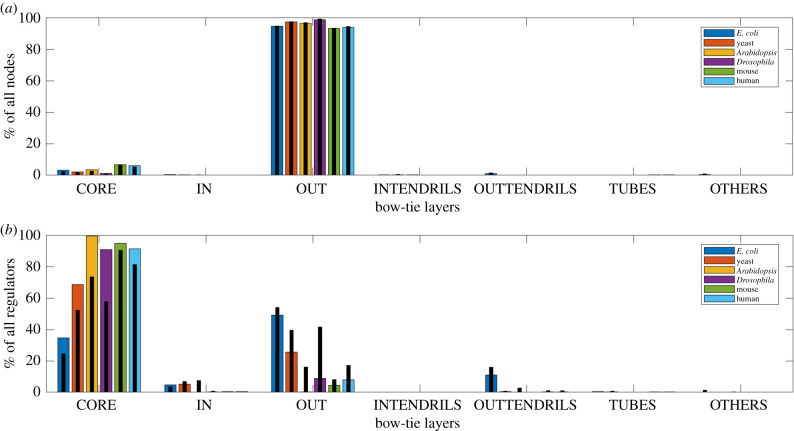
Bow-tie decomposition of GRNs after random addition of 10% edges. (*a*) Average distribution of nodes in different bow-tie layers. (*b*) Average distribution of regulators in different bow-tie layers. The original distribution of nodes and regulators are shown as black bars. The trend of increasing core size with species complexity is still observed.

**Figure 4.**
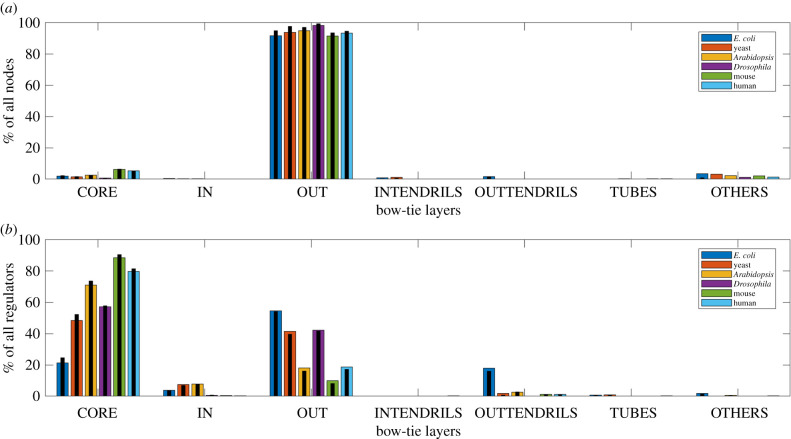
Bow-tie decomposition of GRNs after random deletion of 10% edges. (*a*) Average distribution of nodes in different bow-tie layers. (*b*) Average distribution of regulators in different bow-tie layers. The original distribution of nodes and regulators are shown as black bars. The core sizes are still substantial.

To assess the effects of false positive and missing edges in the extracted GRNs on our observations, we perform sensitivity analysis experiments. In figures 3 and 4, we present the average distribution of nodes and regulators in the different layers from bow-tie decomposition of 1000 GRNs after random addition and deletion of 10% of the original GRN edges, respectively. On addition of edges, the size of the core increases. For *Drosophila* GRN with just one node in the in layer, random edge addition leads to an incomplete bow-tie architecture, with the average number of in nodes, rounded to an integer, being 0. Between species, the generally increasing trend in core size with complexity is still observed. The increase in the core size at the cost of the sizes of layers like the out would depend on factors like the network density and the original layer sizes, governing how a regulator node can now become part of the LSC, which can explain why we observe larger changes for some species in figure 3. On deletion of edges, the core decreases in size, but is still substantially large and the roughly increasing trend in core size with complexity is preserved. There is an increase observed in the size of the others layer. The sensitivity analysis for much larger percentages (25% and 50%) of edge addition and deletion are presented in the electronic supplementary material. Overall, these experiments demonstrate that the observed existence of a bow-tie architecture with an LSC core and the trend of increasing core size with species complexity is quite robust to variations in the quality of the GRN data.

**Figure 5.**
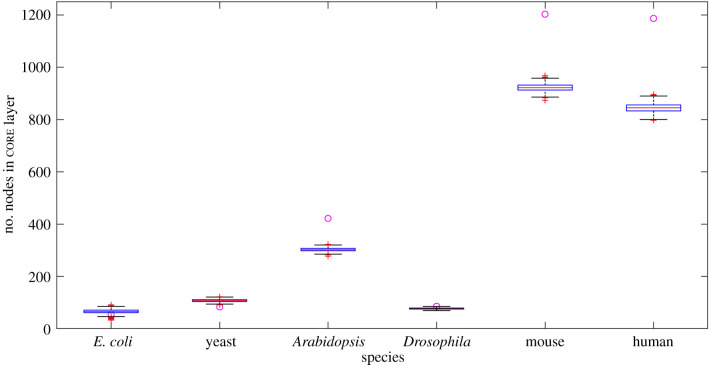
Bow-tie core sizes of similar random networks. Number of nodes in GRN core layer (circle) are compared to those in similar random networks (box plot) for different species. For *E. coli* and yeast, the size of the core is significantly smaller than expected in random networks. For species more complex than yeast, the size of the core is significantly larger than expected in random networks.

Further, to quantify the extent to which the GRN bow-tie architectures are different than what would be expected simply by chance, we compared the bow-tie architectures observed in the empirical GRNs with their randomized counterparts. We looked at the LSC core size in these GRNs and the corresponding sizes in random networks having the same number and degree of nodes (§2.4). Figure 5 shows the LSC core layer sizes of 1000 random networks for every species, along with core size in the original GRNs. We observe that for *E. coli* and yeast, the size of the core is smaller than that expected in similar random networks. As the species complexity increases in eukaryotes beyond yeast, the size of the GRN bow-tie core is larger than expected in random networks. For *Drosophila*, most of the similar random networks do not have a full bow-tie architecture, with 0 nodes in the in layer. Using a *z*-score absolute value threshold of 1.5 as in [15], we can say that the sizes of the bow-tie LSC core in the original GRNs of these species are significantly different from those in random networks. This points to the conclusion that the observed bow-tie architectures are characteristic features of these GRNs differentiating them from random networks of similar size and degree.

## 4. Discussion

### 4.1. Summary of observations

From our results in table 2 and figure 2, we find that a bow-tie architecture with a distinct LSC bow-tie core exists in the GRNs of all six species of varying complexity. We observe that there is a general increase in bow-tie core size, relative to all nodes and all regulators in the GRN, with the complexity of the species. Our sensitivity analysis in figures 3, 4 and electronic supplementary material and comparison with similar random networks in figure 5 show that the bow-tie architectures in these GRNs are characteristic features and cannot be explained just by chance.

Our observations build on and add to the GRN architecture analysis results obtained from prior research. A bow-tie architecture with a distinct LSC core has been previously observed in the dynamical backbone of yeast GRN [4] and in *Arabidopsis* TF–TF networks [15]. However, the authors of [4] did not find a bow–tie architecture in the dynamical backbone of the analysed *E. coli* GRN, with the LSC not much larger than the second LSC. The GRN consisted of 1607 nodes or about 36% of the species total genes. By contrast, with the use of a more complete GRN with greater than 50% of the total genes of the species, we observe a distinct LSC core between in and out layers for the prokaryote *E. coli* and for other more complex eukaryotic species.

We observe an increase in bow-tie relative core size with the complexity of the species, but this increase is not monotonic (figure 2). A possible explanation for these slight variations from the trend of relative core size increase with complexity is variation in the GRN data quality from different data sources. Specifically in figure 2*a*, a larger core size relative to all nodes is observed in *E. coli* than for more complex yeast. There is also a subsequent drop for more complex species *Drosophila*. We believe that the likely cause of this is the incompleteness of the available GRN information in terms of the number of regulators in the extracted GRN. The percentage of regulator nodes out of all network nodes in the extracted GRN, where the corresponding absolute numbers are presented in table 2, is highest for *E. coli* and lowest for *Drosophila*. This might contribute to the observed relatively high and low core sizes with respect to all nodes respectively for these two species. Therefore, we validate the observation that the core becomes larger with complexity by also examining the size relative to all regulators in the GRN in figure 2*b*. Here a clearer increase of core size with complexity is observed. The reason behind the slight drop observed here for *Drosophila* is probably that one of the two sources used by the curators of the *Drosophila* database (§2.1) has a stricter criterion of both binding and transcriptional regulation evidence for interactions. Our sensitivity analysis demonstrates that our results are quite robust to factors related to GRN data quality like incorrect or missing information. It should be noted that this analysed robustness of our observations is not the same as the dynamical property of robustness, which we discuss separately in the next section.

### 4.2. Variation of dynamical properties with complexity

Next, with our observations about the differences in GRN architectures between species, we aim to understand their biological implications. For that purpose, here we use previously proposed associations of dynamical system properties with the bow-tie architecture and specifically its core layer size. This enables us to suggest hypotheses about how some dynamical gene regulatory system properties may have emerged differently with biological complexity.

#### 4.2.1. Robustness and fragility

Robustness of a dynamical system is the property to withstand the effects of external and internal perturbations to maintain its functioning, whereas fragility is the property where the system, robust against expected perturbations, is fragile to some unexpected perturbations [31]. The robustness facilitated by the network bow-tie architecture can follow from the robustness of the strongly connected core [6], due to the presence of multiple paths between any two pairs of core nodes [12]. However, the same system is fragile to sufficient perturbations of the bow-tie core and can be hijacked or disrupted by other processes [8]. In the model of [32], a transition from a random GRN to one with a smaller, denser and segregated core block of nodes, followed by a general decrease in core size, has been observed on increasing the selective evolutionary pressure favouring robustness against noise. In our results, we observe a bow-tie architecture with a LSC core in GRNs of all species, and this architectural feature makes these regulatory systems robust. We hypothesize that the increase in bow-tie core size in more complex species imparts the system increased robustness to perturbations not specifically concentrated in the core, as there are potentially more regulatory paths between any pair of nodes in the bigger LSC core. But this comes at the cost of increased system fragility to specific perturbations to the larger core.

#### 4.2.2. Flexibility

Flexibility refers to the property by which a large number of possible outcomes are supported by a dynamical system [33]. This property describes the internal degrees of freedom in the system [34]. It is pointed out in [8] that a larger bow-tie core providing a wider range of common services increases the system flexibility. Based on our observations in the analysed GRNs, we propose that the larger bow-tie core provides more flexibility in the more complex species. A more complex organism has more cell types, which are involved in more specialized functions, and the larger core structurally has the ability to mediate a larger variety of biological functions. So in these complex organisms the general GRN is observed to have a larger bow-tie core, to increase the flexibility of the regulatory system.

#### 4.2.3. Criticality

Criticality is the property by which a dynamical system tunes to a point or region of marginal stability, and exists at the boundary between the ordered and the chaotic phases [35]. Biological regulatory networks are found to be critical or near critical [35]. This property of criticality allows the system to attain an optimal trade-off between the above-mentioned properties of robustness and flexibility [33]. It is discussed in [17] that a larger bow-tie core might make the system more flexible and shift it more towards the ‘state at the edge of chaos’. Our hypothesis is that a larger GRN core in more complex species moves their gene regulatory systems closer to criticality, and with an increase in flexibility and a decrease in robustness to specific perturbations to the core as discussed before, allows a better robustness–flexibility balance.

#### 4.2.4. Controllability

Another dynamical property of relevance here is controllability. A non-linear dynamical system is defined to be controllable when there is a control path from an undesired attractor state to a desired attractor state under finite perturbations, where attractor states are stable equilibrium states in the phase space [36]. It has been proposed that a larger bow-tie core reduces controllability [8]. Tighter control of the regulatory system may be related to more extreme conditions and less resources [17], which might explain why less complex organisms including bacteria in our analysis have a smaller bow-tie core allowing more rigid control. More complex organisms with more cell types should have more attractor states, as these attractor states of GRNs are considered to correspond to gene expression profiles associated with each cell type [28]. We hypothesize that in such cases, perturbing the regulatory system to move from an undesired attractor to a desired attractor might be more difficult. This reduces the system controllability with complexity, that comes with a larger GRN bow-tie core, as observed for more complex species.

#### 4.2.5. Evolvability

All the properties discussed above are in the context of a short timescale, and a dynamical system long-term property, which the bow-tie architecture is associated with, is evolvability. This is the property by which an organism generates heritable phenotypic variation [37]. At longer timescales, evolvability can be considered as the robustness of lineages to potentially large external or internal changes [8]. Hence the architectural requirements are the same for both robustness and by extension to long time-horizons—evolvability, and these requirements can be met through the bow-tie architecture [6]. It is pointed out in [8] that a larger bow-tie core reduces evolvability, which makes sense if only robustness is considered. However while analysing which systems are more evolvable, we should consider both robustness and flexibility [37], and how these two properties are more optimally balanced through critical behaviour [33,38]. With this consideration, we hypothesize that an increase in GRN core size with species complexity provides increased long-term evolvability in the more complex organisms.

To summarize our hypotheses, an increasing GRN bow-tie core in more complex species gives their gene regulatory systems increased robustness to perturbations not concentrated in the core, but also leads to increased system fragility to specific perturbations to the core. The larger core provides greater flexibility and moves the regulatory system closer to criticality, and gives increased evolvability in the long term. The less complex species have a smaller bow-tie core imparting increased short-term controllability.

We not only put forward hypotheses about how dynamical gene regulatory system properties emerge differently with species complexity, but also are able to suggest a complexity based division between species in terms of these properties. Comparison with random networks similar in size and degree distribution in figure 5 reveals that the LSC core is smaller than expected by chance in *E. coli* and yeast GRNs. Similar results for LSC size were previously observed in GRNs of *B. subtilis* and *E. coli* [18], and yeast [39]. For more complex eukaryotic GRNs, we observe that the bow-tie core size is larger than expected in similar random networks. So it is reasonable to speculate that for prokaryotic bacteria and unicellular eukaryotes living in comparatively more extreme conditions, greater regulatory system controllability is beneficial. On the other hand, for multicellular eukaryotes, increased flexibility at the cost of reduced robustness to specific perturbations and hence behaviour closer to criticality, with subsequently increased long-term evolvability are probably key requirements for the regulatory system. Our work has focused on how the GRN bow-tie architectures in these species have evolved to possibly support these requirements.

## 5. Conclusion

In this paper, we investigate the GRNs of several species and demonstrate the existence of a bow-tie architecture with a distinct LSC core in them. We show that the bow-tie is a characteristic GRN architectural feature. Among the strengths of our work, to our knowledge this is a novel comprehensive bow-tie architecture analysis of GRNs in several species of widely varying complexity. We further observe an increasing trend in relative core size with species complexity and hypothesize how dynamical gene regulatory system properties have emerged differently with complexity. These system properties are tightly coupled with the functioning and pathology of all the organisms. For instance, using the trade-off between robustness and fragility is considered to be a promising direction of cancer therapy [7]. The controllability of the gene regulatory system is very relevant, as cancer cells are considered to be trapped in abnormal attractor states [40]. Understanding how these properties emerge and how they emerge differently between species, can lead to novel systems-based therapy approaches for diseases like cancer. Our work has provided valuable insights into the structural basis of these differences. For instance, the larger bow-tie core size for more complex organisms like human needs to be taken into account in coming up with potential approaches for controlling the regulatory system state. Another possible benefit of our work is that the observed trends from the analysis of GRNs in several well-studied species can provide guiding directions for studies on less-studied or non-model species whose regulatory interaction information is largely incomplete at present.

A limitation of this work is that using other GRN data sources or a different set of GRN extraction criteria could affect our observations. For our analysis, we depend on the information available in existing state-of-the-art biological data sources, with GRN extraction criteria aimed at an optimal ground of comparison. Supported by our sensitivity analysis experiments, we believe our results are quite robust to data quality factors and hence the corresponding possible biological explanations hold merit. As new experimental methods for collecting data on regulatory interactions are developed, more complete and accurate data on regulatory networks for more species should become available. We anticipate that the methods and results presented here will enable more detailed analysis of these data.

Future work could aim at testing the hypotheses proposed in this paper. There exist some approaches applicable in dynamical models for quantifying the system properties discussed here, however, obtaining accurate dynamical models of these general GRNs of different species is a challenging problem on its own [35,41]. Metric definitions on real systems should be standardized. For quantifying properties associated with biological functions, we might need to look for gene functionalities through functional enrichment analysis, or for pathways through pathway enrichment analysis in the bow-tie layers. Our suggested hypotheses about how these properties emerge differently with species complexity could then be verified, and the role of the bow-tie architecture core size difference can be assessed by possible *in vitro* GRN modification experiments. We need to consider other factors, including connectivity within and between different bow-tie layers, that might also control these dynamical properties. However, for verifying the impact of the GRN bow-tie architecture in the proposed relationships, understanding how this architecture governs the network dynamics is of prime importance.

In our work, we only look at the structural relationship of GRN architecture with dynamical gene regulatory system properties, but in future we want to investigate the details of how the network architecture controls the network dynamics. For this we need to understand how the structure of an individual bow-tie layer governs the dynamics associated with that layer, and then possibly extend this to how the global bow-tie architecture controls the global network dynamics, within and between species. Determining how dynamical behaviour associated with specific biological functions or pathways is controlled by the individual layers and the overall bow-tie architecture would provide new and valuable understanding of the functionality of GRNs. In our study, we consider general trends in one direction of either increase or decrease in terms of dynamical properties with general increase of bow-tie core size in more complex species. However, detailed analysis of dynamics could reveal and explain the more complicated nature of these trends [32]. The insights we provide here in our work can be useful for such future dynamical analysis.

## Data accessibility

All data, code and materials are available at https://github.com/gourabghoshroy/Bow-tieGRN.
